# Dual Agent Loaded PLGA Nanoparticles Enhanced Antitumor Activity in a Multidrug-Resistant Breast Tumor Eenograft Model

**DOI:** 10.3390/ijms15022761

**Published:** 2014-02-18

**Authors:** Yan Chen, Xue-Lian Zheng, Dai-Long Fang, Yang Yang, Jin-Kun Zhang, Hui-Li Li, Bei Xu, Yi Lei, Ke Ren, Xiang-Rong Song

**Affiliations:** 1 State Key Laboratory of Biotherapy, West China Hospital, Sichuan University, Chengdu 610041, Sichuan, China; E-Mails: yanzai1112@sina.com (Y.C.); fangdailongtwozero@126.com (D.-L.F.); yyde2013@163.com (Y.Y.); ymyzjk@163.com (J.-K.Z.); 13880286908@163.com (H.-L.L.); xb1990625@126.com (B.X.); caokaijin@163.com (Y.L.); 2 Key Laboratory of Obstetric & Gynecologic and Pediatric Diseases and Birth Defects of Ministry of Education, West China Second University Hospital, Sichuan University, Chengdu 610041, Sichuan, China; E-Mail: zxlian65@aliyun.com; 3 Department of Pharmaceutical Sciences, University of Nebraska Medical Center, Omaha, NE 68198, USA; E-Mail: renkemallee@gmail.com

**Keywords:** multidrug-resistant breast cancer, vincristine, verapamil, PLGA nanoparticles, co-encapsulation

## Abstract

Multidrug-resistant breast cancers have limited and ineffective clinical treatment options. This study aimed to develop PLGA nanoparticles containing a synergistic combination of vincristine and verapamil to achieve less toxicity and enhanced efficacy on multidrug-resistant breast cancers. The 1:250 molar ratio of VCR/VRP showed strong synergism with the reversal index of approximately 130 in the multidrug-resistant MCF-7/ADR cells compared to drug-sensitive MCF-7 cells. The lyophilized nanoparticles could get dispersed quickly with the similar size distribution, zeta potential and encapsulation efficiency to the pre-lyophilized nanoparticles suspension, and maintain the synergistic *in vitro* release ratio of drugs. The co-encapsulated nanoparticle formulation had lower toxicity than free vincristine/verapamil combinations according to the acute-toxicity test. Furthermore, the most effective tumor growth inhibition in the MCF-7/ADR human breast tumor xenograft was observed in the co-delivery nanoparticle formulation group in comparison with saline control, free vincristine, free vincristine/verapamil combinations and single-drug nanoparticle combinations. All the data demonstrated that PLGANPs simultaneously loaded with chemotherapeutic drug and chemosensitizer might be one of the most potential formulations in the treatment of multidrug-resistant breast cancer in clinic.

## 1. Introduction

Breast cancer is the most common cancer in women, with more than 1,300,000 cases and 450,000 deaths each year worldwide [1]. Despite considerable advances in early detection as well as therapeutic strategies with surgery, radiotherapy, and chemotherapy, the mortality rates in breast cancer patients have remained relatively unaffected over the span of three decades [2]. A major obstacle to a more effective cure for this disease is put down to multidrug resistance (MDR) [3] of cancer cells predominantly mediated by overexpressing proteins belonging to the ATP binding cassette (ABC) transporter superfamily. The MDR proteins are responsible for energy dependent efflux of drugs, resulting in less likeliness to accumulate therapeutically relevant doses of chemotherapeutics in cancer cells. Among these proteins, P-glycoprotein (Pgp), encoded by the MDR1 (ABCB1) gene is the first ever identified ABC and the most well studied [4].

Several Pgp inhibitors have been explored over the last four decades to overcome MDR in cancer [5,6]. The first generation of ABC blockers such as verapamil, cyclosporine A and quinidine are the most widely investigated probably because they are drugs already approved by the regulatory agencies for other uses [7] and easy to be clinically evaluated as ABC inhibitors for new intended uses. The clinical studies on breast cancer have shown that a combination of the antitumor drug vincristine (VCR) together with Pgp blocker verapamil (VRP) can enhance antitumor activity [8], in which both were traditional formulations. However, the potentiation of neurotoxicity and hematotoxicity was observed [9]. This highlights the need to develop safe and effective drug delivery systems against breast cancers with MDR.

Novel nano-sized formulations including nanoparticles, liposomes, micelles have the potential to solve the problems of toxicity and lack of efficacy, some of which have been approved for clinical use (Abraxane, Doxil, Genexol-PM) [10].The toxicity of the chemotherapeutic drug or chemosensitizer encapsulated into nano-sized formulations could be reduced because the drug could not exert its activity when sequestered in preparations during bloodstream circulation [11]. Moreover, nano-sized formulations have been shown to enhance therapeutic efficacy of anticancer drugs by increasing drug exposure in the tumor due to the prolonged circulation times of the drugs, and by preferential accumulation of the anticancer drugs as a result of the EPR effect in the tumor [12].

Given these above advantages, our approach was to develop VCR and VRP-coencapsulating PLGA nanoparticles (VCR-VRP-PLGANPs) with less toxicity and enhanced efficacy on multidrug-resistant breast cancers. Our previous findings demonstrated that administration sequence of anticancer drug and chemosensitizer was critical for maximal therapeutic efficacy, and the highest *in vitro* reversal efficacy has been proved to be achieved by the simultaneous administration of VCR and VRP [13]. Based on these results, VCR-VRP-PLGANPs suspension has been successfully prepared, and verified to be slightly more cytotoxic than free VCR/VRP combination on multidrug-resistant human breast carcinoma cell line MCF-7/ADR and be similar to single-drug nanoparticles combinations in *in vitro* reversion activity of multidrug resistance on MCF-7/ADR cells [14]. In this study, we aimed to further develop lyophilized VCR-VRP-PLGANPs for long-term storage, and assess whether nanoparticle encapsulation would reduce the *in vivo* toxicity of free VCR/VRP combination and determine if the co-encapsulated nanoparticles could improve the antitumor efficacy in the human MCF-7/ADR multidrug-resistant breast tumor xenograft model.

## 2. Results and Discussion

### 2.1. *In Vitro* Combination Effects of Free VCR and VRP

The *in vitro* cytotoxicity studies were first conducted with VCR/VRP combinations on MCF-7/ADR cells to determine whether VRP could reverse the drug resistance and what was the optimum dose for VRP to achieve strong synergism with VCR. As shown in Table 1, all the combination index (*CI*) values at *IC*_50_ were less than 0.9 when VRP was set at different concentration, and an obvious synergistic interaction of VCR and VRP was demonstrated. Moreover, VRP achieved concentration-dependent enhancement of the sensitivity of MCF-7/ADR cells to VCR. When VRP was set at the concentration of 10 μM, the reversal index reached 130 in this combinational group and the *IC*_50_ value of VCR/VRP against MCF-7/ADR cells was equal to that of single VCR against MCF-7 cells. Thus, 10 μM VRP was considered to be enough to recover the sensitivity of MCF-7/ADR cells to VCR.

The cell growth inhibition percentage of 0.02 μM VCR in combination with 10 μM VRP was 57.95% ± 0.43% in MCF-7/ADR cells, in which the molar ratio of VCR/VRP was 1:500. Nanoparticles encapsulation might enhance the antitumor activity of drugs [15,16], hence VCR/VRP at 1:500 molar ratio was selected to be entrapped into PLGA nanoparticles and be further investigated.

### 2.2. Preparation and Characterization of Lyophilized VCR-VRP-PLGANPs

The effects of different lyoprotectants on conservation of prepared VCR-VRP-PLGANPs were investigated on the basis of the appearance of dried powders and the re-dispersed time of lyophilized particles by distilled water. Using lactose or mannital (6% *w*/*v*) as lyoprotectant during freeze drying, the good appearance of the lyophilized preparation was observed, whereas the lyophilized particles could be re-dispersible within 20 s using lactose, which was much faster than using mannital. Lactose was one of the widely used cryoprotectants [17,18], thus 6% lactose was selected to protect VCR-VRP-PLGANPs during lyophilization.

Table 2 illustrated the physico-chemical related properties of VCR-VRP-PLGANPs before and after lyophlization with 6% lactose as lyoprotectant. Statistical analysis showed no significant differences in size distribution, zeta potential and EE% before and after lyophilization (*p* > 0.05), clearly indicating good conservation during the lyophilization process.

**Figure 1.**
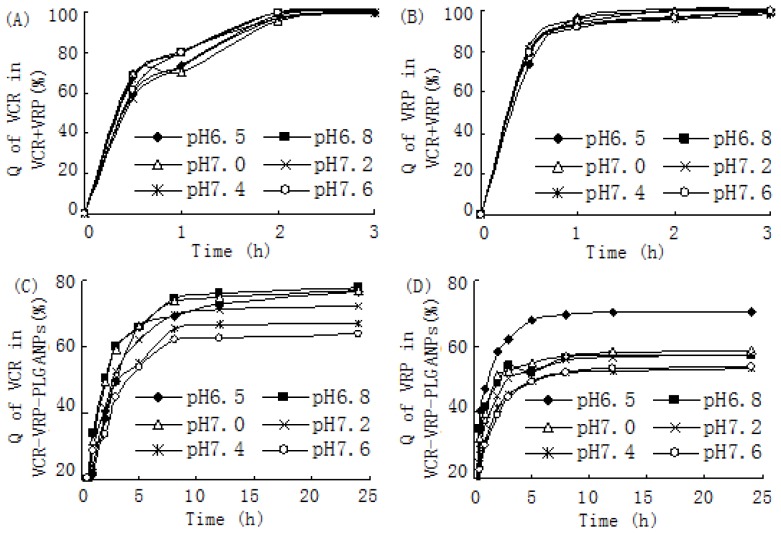
*In vitro* release curves of VCR and VRP by dialysis method in phosphate buffers with various pH values. (**A**) The accumulative release quantity (Q) of VCR from free VCR/VRP combinations within 3 h; (**B**) The accumulative release quantity (Q) of VRP from free VCR/VRP combinations within 3 h; (**C**) The accumulative release quantity (Q) of VCR from VCR-VRP-PLGANPs within 24 h; (**D**) The accumulative release quantity (Q) of VRP from VCR-VRP-PLGANPs within 24 h.

VCR-VRP-PLGANPs before and after lyophlization with 6% lactose as lyoprotectant. Statistical analysis showed no significant differences in size distribution, zeta potential and EE% before and after lyophilization (*p* > 0.05), clearly indicating good conservation during the lyophilization process. In this study, several phosphate buffer solutions with different pH values from 7.6 to 6.5, simulating the environment of blood and microenvironment of tumor cells [19], were selected to be *in vitro* release media. Figure 1A,B represented the release profiles of VCR and VRP from free VCR/VRP combinations, respectively. Almost all the drugs had released into the outer medium from the dialysis bag within 2 h, which demonstrated that using a dialysis bag of this kind was suitable to investigate the *in vitro* release profiles of VCR-VRP-PLGANPs because no retention or absorption of drugs was found.

VCR-VRP-PLGANPs maintained the synergistic drug ratio for *in vitro* release, and release of VCR and VRP from nanoparticles showed a biphasic behavior comprising an initial phase release of drugs close to the surface of nanoparticles during the first 2 h. This was followed by an extended controlled release phase of several hours (post 2 h). VCR-VRP-PLGANPs showed a release of 60%–80% in phosphate buffer from pH 6.5 to 7.6 after 24 h (Figure 1C,D). The prolonged and slow release of VCR and VRP from VCR-VRP-PLGANPs after 2 h can be explained in terms of a very slow diffusion of drugs from the inner core matrix of the nanoparticles [14]. Various release models including Weibull, Niebergull, Higuchi, Hixson-Crowell, Noexponential and Monoexpotential were applied on the release data. The release kinetics were concluded to follow the Weibull formula as indicated by the highest value of *R* (*R* > 0.9) observed in this case. According to the Weibull formula, the time of 50% drug release from nanoparticles (*T*_0.5_) was calculated and the results were presented in Table 3.

In the light of ANOVA, there were no differences on *T*_0.5_ values of VCR and VRP releasing from free VCR/VRP combinations in different pH release media (*p* > 0.05), while the release of VCR and VRP from VCR-VRP-PLGANPs was significantly faster in pH 6.5–7.0 release media than in pH 7.2–7.6 release media (*p* < 0.05). This kind of faster release in lower pH would facilitate the antitumor therapy with VCR-VRP-PLGANPs [20,21].

### 2.3. Acute Toxicity Test

Up-and-down procedure is an alternative to the *LD*_50_ acute toxicity test, which only requires a fewer animals to achieve similar accuracy as the *LD*_50_ test [22] and is likely suitable for drugs with rapid lethal toxicity. No death was found in mice within 2 h after intravenous administration of free VCR with a dose up to 10 g/kg, thus the acute toxicity of VCR was not determined using the up-and-down procedure. As described in Table 4, *LD*_50_ values of both VCR and VCR/VRP combinations were found to be similar (*p* < 0.05), thus it may be predicted that the introduction of VCR would not increase the intrinsic toxicity of VRP and the combination of VCR and VRP were feasible. Additionally, all the nano-formulations were far less toxic than the free drugs in mice following intravenous administration, possibly ascribing to the sustained release of nanoparticles during blood circulation. The result demonstrated that these nanoparticles can be exploited for potential therapeutic application.

### 2.4. *In Vivo* Antitumor Effects against the MCF-7/ADR Xenograft

**Figure 2.**
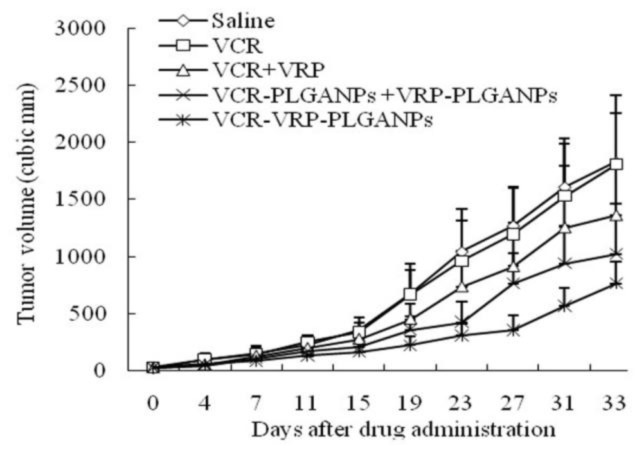
*In vivo* antitumor effects of various treatment groups against MCF-7/ADR tumor xenograft in BALB/c nude mice (*n* = 6). The mice were treated via tail vein injections with saline control, free VCR, free VCR/VRP combination, single-drug nanoparticle combinations and co-encapsulated nanoparticles every three days. The doses of vincristine and quercetin given were 0.05 and 1.25 mg/kg, respectively (1:500 VCR/VRP molar ratio).

The *in vivo* antitumor effects of co-delivery VCR-VRP-PLGANPs against MCF-7/ADR human breast tumor xenograft were investigated by comparing with saline control, free vincristine, free VCR/VRP combinations and single-drug nanoparticle combinations. As seen in Figure 2, the tumor growth of the VCR-VRP-PLGANPs group was the slowest. After treating ten times, VCR-VRP-PLGANPs exhibited a significant antitumor activity in inhibiting tumor progress compared with saline control. Statistically significant difference in the inhibition rate of tumor mass (Table 5) was observed between the co-encapsulated nanoparticles and the other study groups (*p* < 0.05). Free VCR monotherapy was not statistically different from the saline control (*p* > 0.05), though the combination of VRP did enhance the antitumor efficacy of VCR, which was consistent with the clinical study on breast cancer [8]. Co-encapsulation of VCR and VRP into PLGA nanoparticles further amplified the antitumor effect of VCR/VRP combinations, probably the result of the increased accumulation of nanoparticles in tumor tissue and the fast release in the weakly acidic internal environment of tumor cells. The inhibition efficiency of VCR-VRP-PLGANPs against MCF-7/ADR xenograft was only 64%, mainly because the dose of VCR was a great deal lower than that reported by other literatures [11,23]. Thus, to further increase the dose would likely achieve better therapeutic efficacy.

## 3. Experimental Section

### 3.1. Materials

VCR (purity 98%) was purchased from Huanye Pharmaceutical Co., Ltd. (Guangzhou, China). VRP was obtained from Central Pharmaceutical Co., Ltd. (Tianjin, China). Adriamycin (ADR) was bought from Zhejiang Hisun Pharmaceutical Co., Ltd. (Zhejiang, China). PLGA 75:25 DL (molecular weight, 15 kDa) was purchased from Shandong Institute of Medical Devices Department of medical polymers Shandong institute, China. PVA205 (88% of hydrolyzation degree, 500 of polymerization degree) was purchased from Kuraray Co., Ltd., Shanghai, China. All reagents were of analytical grade and were used without further purification.

### 3.2. Cell Culture and *in Vitro* Cytotoxicity Assay of Free Drugs

MCF-7/ADR was developed from the parental drug-sensitive MCF-7 cells, obtained from the American Type Culture Collection, by stepwise selection for resistance with increasing concentration of ADR and maintained in the presence of 1 μg/mL of ADR. The cells were grown in RPMI 1640 containing 10% fetal bovine serum and 1% penicillin/streptomycin in a humidified atmosphere containing 5% CO_2_ at 37 °C. MCF-7 cells were cultured in the same way to MCF-7/ADR except the absence of ADR.

*In vitro* cytotoxicity was assessed by MTT assay [14]. Briefly, MCF-7 and MCF-7/ADR cells were seeded at 3500 and 6000 cells/well in 96-well plates, respectively. Cells were incubated for 24 h to allow adherence to the cell culture plates before treatment with serial dilutions of either single drugs (VCR, VRP) or drug combinations (VCR in combination with VRP at different concentrations of 2.5, 5, 10 and 25 μg/mL) for 48 h. Subsequently, 20 μL of MTT reagent (5 mg/mL) was added to each well and incubated with the cells for additional 4 h. The cell culture medium was aspirated, and 150 μL of DMSO was added to each well. The 96-well plates were shaken for 10 min to solubilize the formazan crystals and subsequently were read on the ELISA plate reader (Bio-Rad, Microplate Reader 550, Hercules, CA, USA) with an absorbance wavelength of 570 nm. Cell survival percentage was calculated from the absorbance readings as a percentage of the control. All assays were performed in triplicate.

The *IC*_50_ values of VCR solution or VCR/VRP combinations were calculated according to a series of dose-response data using GraphPad Prism software.

The reversal index of VRP was calculated from Equation.

(1) $$\text{Reversal index}=\frac{IC_{50.\text{VCR}}}{IC_{50.\text{VCR}/\text{VRP}}}$$

where *IC*_50.VCR_ was the *IC*_50_ value of solution against MCF-7/ADR, *IC*_50.VCR/VRP_ was the *IC*_50_ value of VCR/VRP combinations against MCF-7/ADR.

Drug combination analyses were performed using CompuSyn (ComboSyn, Inc., New York, NY, USA). The CompuSyn software generates the combination index (*CI*) value for a particular combination of VCR/VRP based on the cell-survival data from the MTT assay, whereby additivity, synergy and antagonism were reflected by *CI* values of 0.9–1.1, <0.9 and >1.1, respectively [24].

### 3.3. Preparation and Characterization of Lyophilized VCR-VRP-PLGANPs

VCR-VRP-PLGANPs were prepared according to our previous report [13]. In brief, PLGA (75:25, 15,000, 80 mg), VCR (50 μg), and VRP (10 mg) were dissolved into 1.5 mL of acetone–dichloromethane (0.8/1, *v*/*v*), which formed an organic phase. The organic phase was emulsified with 4 mL of pH 7.4 phosphate buffered solution containing PVA 205 (2%, *w*/*v*) by probe sonication at 50 W for 30 s in ice bath. The organic solvent was then rapidly evaporated under reduced pressure at 37 °C. The obtained colloidal suspension was further ultracentrifuged for 1 h at 4 °C at 223,000× *g* (Optima MAX-E Ultracentrifuge, Beckman Coulter Inc., Brea, CA, USA). The sediments were resuspended with water to achieve VCR-VRP-PLGANPs. The achieved nanoparticles were frozen at −40 °C overnight. Lyophilization was preceded by using a Virtis Advantage XL-70/Freeze Dryer (SP Scientific, New York, NY, USA) under vacuum (100 mTorr) with condenser temperature of −48 °C. Freeze drying was performed using different lyoprotectants including sucrose, glucose, lactose and mannitol with various concentrations (*i.e.*, 1%, 2%, 4% and 6% (*w*/*v*)). After lyophilization for 36 h, the dried samples were observed on the appearance and then reconstituted in distilled water, and the re-dispersed time of lyophilized particles was recorded. The size and zeta potential of re-dispersed particles were measured using a Zetasizer Nano ZS90 (Malvern Instruments Ltd., Worcestershire, UK). The entrapment efficiencies of VCR and VRP were determined according to methods reported in our previously published articles [25]. All measurements were performed in triplicate. For the preparation of nanoparticles containing only VCR or VRP, the same procedure was followed, except that only the stated drug was included in the preparation.

*In vitro* release of VCR and VRP from lyophilized VCR-VRP-PLGANPs was determined in series of phosphate buffers with various pH values (6.5, 6.8, 7.0, 7.2, 7.4 and 7.6) by dialysis bag method using dialysis membrane with a molecular weight cut off of 3500 Da. An accurate volume (1 mL) of re-dispersed VCR-VRP-PLGANPs was placed into the dialysis bag which was tied tightly at both the ends and dipped in the dissolution medium (20 mL), which was shaken in a constant temperature shaker (Taicang biochemical instrument industry, Jiangsu, China) at 70 rpm at 37 ± 1 °C. At regular time intervals, 0.5 mL of the release medium was withdrawn at pre-set time intervals (0.25, 0.5, 1, 2, 4, 8, 12 and 24 h) and replaced by an equal volume of fresh medium. The samples were analyzed by HPLC [14]. *In vitro* release of VCR and VRP from free VCR/VRP combinations was carried out in the same way. The data were expressed as % cumulative drug released (Q) *versus* time plots for the free drug and for the drug-loaded PLGA nanoparticles.

### 3.4. Animal Studies

The studies were approved by the Institutional Animal Care and Use Committee of Sichuan University, and the procedures followed were in accordance with National Institutes of Health Guide for the Care and Use of Laboratory Animals. The animals were under humane care throughout the studies and were housed in micro-isolator cages with free access to food and water.

### 3.5. Acute Toxicity Study in Mice

The acute toxicity study was carried out on the up-and-down method. Several female Kunming mice weighing 18 to 22 g were used in this study. *LD*_0_ and *LD*_100_ values were firstly determined. Four mice were grouped to test each dose. If all the mice survived, the dose for the next group was increased. If all died, the dose was decreased. *LD*_100_ was the upper dose close to that with which 3 mice died. *LD*_100_ was the lower dose proximate to that with which 1 mouse died. The following main test proceeded. Single mouse was dosed in sequence at 48 h intervals. Using the default progression factor and *LD*_0_ and *LD*_100_ values, doses to be administered were calculated using AOT425StatPgm program. Initial dose of *LD*_0_ and maximal dose of *LD*_100_ were selected. Each mouse was observed for 24 h before dosing the next animal, and all the surviving mice were observed individually once a day for 14 days. *LD*_50_ was finally calculated using AOT425StatPgm program.

### 3.6. *In Vivo* Efficacy Study

Tumors were established in BALB/c nude mice (4 weeks old) by a single subcutaneous injection of 5 × 10^7^ MCF-7/ADR cells in the upper back area. Tumor progression was monitored by caliper measurements of the tumors along the length and width twice a week. Tumor volumes were calculated by the following formula:

(2) $$\text{Tumor volume}=\frac{\text{length}\times {\text{width}}^{2}}{2}$$

When the tumor size reached approximately 100 mm^3^, the mice were randomly divided into 5 groups of 6 animals each, and received intravenous injection of saline control, free VCR, free VCR/VRP combination, single-drug nanoparticle combinations (VCR-PLGANPs + VRP-PLGANPs) and co-delivery VCR-VRP-PLGANPs every 3 days, respectively. The doses of VCR and VRP were 0.05 and 1.25 mg/kg, respectively, among which the molar ratio of VCR/VRP was 1:500. Tumor size and body weight of the mice was monitored periodically. All the mice were euthanized after treatment for 10 times.

### 3.7. Statistical Analysis

All statistical analyses were performed using the Statistical Product and Service Solutions software (SPSS V 13.0, SPSS Inc., Chicago, IL, USA). Data were analyzed by One-Way ANOVA and Dunnett’s multiple comparison tests. *p* values of < 0.05 were considered to be statistically significant.

## 4. Conclusions

The optimal molar ratio of VCR to VRP coencapsulated into PLGA nanoparticles was founded to be 1:500 by *in vitro* cytotoxicity assay. The lyophilized PLGA nanoparticles, simultaneously loaded with VCR and VRP at the designed mass ratio, was successfully constructed using 6% lactose as lyoprotectant. The dual drug-loaded nanoparticles, with synergistic *in vitro* release ratio of drugs, presented slightly fast release in the acidic environment similar to internal environment of tumor cells. The combination of VCR and VRP would not increase the intrinsic toxicity of VRP and coencapsulation into nanoparticles would significantly reduce the toxicity, indicating that co-delivery nanoparticles formulation of VCR/VRP combinations was feasible. The *in vivo* test demonstrated that the co-encapsulation of VCR and VRP into PLGA nanoparticles at synergistic ratio showed good antitumor efficacy in multidrug-resistant MCF-7/ADR human breast tumor xenograft models. Therefore, the coencapsulated PLGA nanoparticles formulation of VCR and VRP had the potential to be developed further into a possible clinical treatment option for multidrug-resistant breast cancer.

## Figures and Tables

**Table 1.** Cytotoxicities of VCR, VRP, VCR/VRP against drug-sensitive cells and drug-resistant cells (*n* = 3).

Cells	Drugs	*IC*_50_ values (nM)	Reversal index	*CI* values at *IC*_50_
MCF-7	VCR	8.79 ± 0.63	–	
MCF-7/ADR	VCR	1202.55 ± 292.51	–	
	VRP	98214.10 ± 4907.65	–	
	VCR + 5 μM VRP	16.25 ± 3.47 A	74.00	0.0136
	VCR + 10 μM VRP	9.21 ± 2.06 A	130.59	0.00776
	VCR + 20 μM VRP	7.69 ± 2.93 A	156.34	0.0066
	VCR + 50 μM VRP	8.34 ± 2.71 A	144.16	0.00744

A *p* < 0.05, compared to free VCR.

**Table 2.** Physico-chemical characteristics of VCR-VRP-PLGANPs before and after lyophilization (*n* = 3).

Physico-chemical characteristics	Drugs	Data (Mean ± SD)	

		Before lyophilization	After lyophilization
Size (nm)		111.40 ± 2.40	120.80 ± 8.20
PDI		0.062 ± 0.023	0.074 ± 0.015
Zeta potential (mV)		−0.75 ± 0.12	−0.63 ± 0.24
EE (%)	VCR	55.35 ± 4.22	50.37 ± 6.39
	VRP	69.47 ± 5.34	67.66 ± 4.51

**Table 3.** The *T*_0.5_ values of VCR and VRP *in vitro* release calculated according to the Weibull formula.

pH of medium	VCR + VRP		VCR-VRP-PLGANPs	

	VCR	VRP	VCR	VRP
6.5	0.54 ± 0.05	0.24 ± 0.07	2.89 ± 0.37	0.42 ± 0.15
6.8	0.43 ± 0.15	0.20 ± 0.03	2.92 ± 0.13	0.68 ± 0.17
7.0	0.50 ± 0.10	0.24 ± 0.01	2.83 ± 0.29	0.66 ± 0.12
7.2	0.37 ± 0.15	0.17 ± 0.03	4.17 ± 0.25	1.42 ± 0.44
7.4	0.53 ± 0.25	0.18 ± 0.09	5.30 ± 0.26	2.83 ± 0.30
7.6	0.31 ± 0.16	0.18 ± 0.04	7.46 ± 0.63	4.45 ± 0.51

**Table 4.** The *LD*_0_, *LD*_50_ and *LD*_100_ values of VCR-VRP-PLGANPs in comparison with VRP, free VCR/VRP combinations, VRP-PLGANPs and single-drug nanoparticle combinations.

Drugs	Dose (mg/kg)		

	*LD*_0_	*LD*_100_	*LD*_50_ (Mean ± SD)
VRP	2.50	16.00	5.99 ± 0.83
VCR/VRP	2.18	13.00	4.93 ± 0.93
VRP-PLGANPs	3.83	12.66	8.44 ± 1.11 A
VCR-PLGANPs + VRP-PLGANPs	3.56	13.95	7.85 ± 0.71 B
VCR-VRP-PLGANPs	4.18	14.67	8.52 ± 1.54 B

A *p* < 0.05, compared to free VRP group;

B *p* < 0.05, compared to free VCR/VRP combinations group.

**Table 5.** The tumor mass of MCF-7/ADR bearing nude mice and the inhibition efficiency after different treatments (Mean ± SD) (*n =* 6).

Group	Tumor mass (g)	Inhibition efficiency (%)
Saline	0.89 ± 0.15	–
VCR	0.83 ± 0.21	6.74
VCR/VRP	0.62 ± 0.19	30.34
VCR-PLGANPs + VRP-PLGANPs	0.47 ± 0.13 A	47.19
VCR-VRP-PLGANPs	0.32 ± 0.05 AA	64.04

A *p* < 0.05;

AA *p* < 0.01, compared to saline control.
