# Correction: Yan Chen, et al. Dual Agent Loaded PLGA Nanoparticles Enhanced Antitumor Activity in a Multidrug-Resistant Breast Tumor Eenograft Model. *Int. J. Mol. Sci.* 2014, *15*, 2761–2772.

**DOI:** 10.3390/ijms17081233

**Published:** 2016-07-29

**Authors:** Yan Chen, Xue-Lian Zheng, Dai-Long Fang, Yang Yang, Jin-Kun Zhang, Hui-Li Li, Bei Xu, Yi Lei, Ke Ren, Xiang-Rong Song

**Affiliations:** 1 State Key Laboratory of Biotherapy, West China Hospital, Sichuan University, Chengdu 610041, Sichuan, China; yanzai1112@sina.com (Y.C.); fangdailongtwozero@126.com (D.-L.F.); yyde2013@163.com (Y.Y.); ymyzjk@163.com (J.-K.Z.); 13880286908@163.com (H.-L.L.); xb1990625@126.com (B.X.); caokaijin@163.com (Y.L.); 2 Key Laboratory of Obstetric & Gynecologic and Pediatric Diseases and Birth Defects of Ministry of Education, West China Second University Hospital, Sichuan University, Chengdu 610041, Sichuan, China; zxlian65@aliyun.com; 3 Department of Pharmaceutical Sciences, University of Nebraska Medical Center, Omaha, NE 68198, USA; renkemallee@gmail.com

The authors wish to make a change to their published paper [1]. The title should read: “Dual Agent Loaded PLGA Nanoparticles Enhanced Antitumor Activity in a Multidrug-Resistant Breast Tumor Xenograft Model”. The authors apologize for any inconvenience the change may cause.

The change does not affect the scientific results. The manuscript will be updated and the original will remain online on the article webpage.
